# The Art, Microbial Quality, Safety, and Physicochemical Characteristics of *Jikita*: A Traditional Ethiopian Fermented Beverage

**DOI:** 10.1155/2024/6698831

**Published:** 2024-07-16

**Authors:** Semira Kemal, Anbessa Dabassa Koricha

**Affiliations:** College of Natural Sciences Department of Biology Jimma University, Jimma, Ethiopia

**Keywords:** cereal-based beverage, cultural significance, Oromo ethnic groups, physicochemical composition, proximate analysis, thick texture

## Abstract

*Jikita* is a traditional fermented beverage popular among the Oromo ethnic groups in Ethiopia. It is made from cereal and has a high alcohol content and thick texture. *Jikita* is widely consumed in the Western Oromia region of Ethiopia and holds significant socioeconomic and cultural importance. However, there is limited knowledge regarding the microbial quality and safety of *Jikita*, as well as its physicochemical and proximate composition. This study is aimed at assessing the current state of *Jikita* production and consumption. Samples were collected from two districts in the West Shewa Zone, where *Jikita* is most prevalent. A survey was conducted to gather information on production methods, sanitary conditions, ingredient composition, and the socioeconomic importance of *Jikita*. The samples were then analyzed for microbial counts, identification, and dynamics, as well as for pH, titratable acidity (TA), moisture, total solid, alcohol, carbohydrate, fat, and protein contents. The results showed that the majority of producers and sellers were middle-aged women who did not use protective clothing. Microbial counts revealed that the levels of aerobic mesophilic bacteria, yeasts, and lactic acid bacteria (LAB) were below the WHO/FDA standards, and no *Salmonella* spp. were detected. The samples exhibited varying pH, TA, moisture, total solid, alcohol, carbohydrate, fat, and protein contents. The microbial dynamics during fermentation showed that different groups of bacteria and yeasts dominated different stages. The overall microbial quality of *Jikita* was indicative of spoilage microorganisms. However, the duration of fermentation inhibited the growth of pathogenic microorganisms and extended the shelf life of the product to more than 2 months. This study provides valuable insights into traditional fermented beverages and their implications for public health. It also suggests the need for improved hygiene practices and quality control measures in *Jikita* production and consumption.

## 1. Background of the Study

Fermented foods and beverages are widely consumed in many parts of the world, particularly in Africa, where they hold great historical and cultural significance. Fermentation is a process that involves the conversion of carbohydrates into alcohol or organic acids by microorganisms, including bacteria, yeasts, and molds [1, 2]. Fermentation not only enhances the flavor, texture, shelf life, nutritional value, and safety of food and beverages, but it can also have positive effects on consumers' health due to the presence of probiotics. Probiotics are live microorganisms that provide health benefits when consumed [3, 4]. This is achieved by promoting digestive health, improving the bioavailability of nutrients, supporting immunity, reducing the risk of cardiovascular diseases, improving metabolic health, reducing muscle soreness, enhancing mood, and promoting gut health. In Africa, fermented foods and beverages commonly reflect the diversity and identity of different regions and ethnic groups, as they are typically made using locally available raw materials and traditional preparation methods.

Microbial fermentation plays a crucial role in the production of traditional fermented foods and beverages. The presence of yeasts and lactic acid bacteria (LAB) during fermentation helps create the desired sensory attributes, such as aroma, taste, and texture. Therefore, it is important to have a good understanding of traditional household processing methods as well as the types and microbial loads involved to ensure the quality and safety of these products while preserving their cultural authenticity.

Ethiopia is a country with a rich and diverse culture of fermented foods and beverages, which have been consumed for thousands of years. Some of the most popular fermented products in Ethiopia include *Enjera*, a sourdough flatbread made from *Tef* and other cereals; *Tej*, a honey wine flavored with *Gesho* leaves; *Tella*, a beer made from barley, maize, sorghum, or millet; and *Borde*, a lactic acid fermented beverage made from cereals and legumes [1, 5]. These products not only provide energy, protein, vitamins, minerals, and probiotics, but they also play an important role in social and religious ceremonies, hospitality, and income generation [3].

However, despite the popularity and significance of fermented foods and beverages in Ethiopia, there is still a lack of scientific documentation and characterization for some of them, particularly those consumed by specific ethnic groups or in certain regions. One such product is *Jikita*, a thick alcoholic beverage made from sorghum and maize, which is widely consumed by the Oromo people in the western part of the country. *Jikita* is known for its high alcohol content, ranging from 10% to 25%, and its long shelf life, lasting up to 10 days. However, the nutritional composition, microbiological profile, physicochemical properties, and sensory attributes of *Jikita* have not been reported in the literature.

Therefore, the objective of this study was to investigate the nutritional, microbiological, and physicochemical of *Jikita* and compare them with those of other Ethiopian fermented beverages, such as *Tej*, *Tella*, and *Borde*. Additionally, this study is aimed at identifying the microorganisms involved in the fermentation process of *Jikita*. The findings of this study will contribute to the scientific understanding and knowledge of *Jikita*, as well as the preservation and promotion of the traditional fermentation practices and culture of the Oromo people.

## 2. Materials and Methods

### 2.1. Sample Collection and Processing Techniques

Samples of *Jikita* were collected from two districts in the West Shewa Zone to evaluate their microbial quality, safety, physicochemical characteristics, and nutritional content. Data were gathered through questionnaires administered to both home producers and vendors. The questionnaires used in this study were rigorously validated by a panel of experts in the field of microbiology and fermentation. They provided critical feedback on the content, structure, and clarity of the questions. Additionally, a pilot study was conducted with a select group of participants to ensure the reliability and validity of the questionnaires. The results of the expert validation and pilot testing were used to refine the questionnaires further. The microbial dynamic analysis of *Jikita* samples was conducted at Jimma University, Department of Biology research laboratory. The microbiology of the samples was analyzed using standard methods, and microbial counts were reported as log CFU/milliliter.

### 2.2. Microbial Enumeration and Isolation

The study utilized various techniques to count and identify different types of microorganisms present in a *Jikita* sample. These techniques involved using diverse culture media, incubation conditions, and morphological characteristics to differentiate among microorganisms. Additionally, a reference was used for each organism's identification technique, such as aerobic mesophilic bacteria were identified using the method described by Wei, Hwang, and Chen [6], LAB [7], coliforms [6], staphylococci and spore-forming bacteria [8], Enterobacteriaceae, and yeast and molds [9]. Furthermore, these techniques enabled the researchers to identify the organisms present in the sample and to estimate the number of organisms.

### 2.3. Techniques Used for Microbial Analysis

To identify aerobic mesophilic bacteria, 10–15 colonies with distinct morphological features such as differences in color, size, and shape were randomly selected from countable plates. These colonies were then aseptically transferred into a tube containing 5 mL of nutrient broth. The inoculated cultures were incubated at a temperature of 32°C for 24 h. After incubation, the cultures were purified through repeated plating and then preserved on agar slants at a temperature of 4°C. Finally, the organisms obtained were classified at the genus and family levels using Yousef and Carlstrom [10] bacterial classification manual.

### 2.4. Cell Morphology

Various microbiological techniques were used to study the cell morphology of the isolates. This included staining with dyes to observe their cell shape and Gram reaction (as described by [11]), checking their motility [12], and evaluating endospore formation to further characterize the isolates.

### 2.5. Biochemical Tests

Various biochemical tests were utilized to distinguish and classify isolated bacteria based on their metabolic and enzymatic activities. Each test employed distinct references and protocols for identification, such as the KOH test [13], the oxidation-fermentation test [14], the catalase test [15], the oxidase test [16], and the carbohydrate fermentation test.

### 2.6. Isolation and Characterization of Pathogens

The harmful microorganisms such as *Staphylococcus aureus* and *Salmonella* sp. were isolated and identified from *Jikita* samples. Various methods, including the use of selective and differential media, biochemical tests (such as triple sugar iron agar (TSIA), lysine iron agar (LIA), urea agar (UA), Simmons citrate test (SCA), and sulfur indole motility (SIM)), and staining techniques (such as the coagulase test), were utilized to characterize the bacteria based on their morphology, growth patterns, and metabolic functions.

### 2.7. Characterization of LAB

Characteristics of LAB were determined using pure cultures of the isolates and analyzed through various methods, including morphological, biochemical, and physiological tests. Morphological tests examined the shapes of the colonies and cells of the isolates. Biochemical tests were conducted to check the Gram reaction, catalase activity, KOH solubility, motility, O/F reaction, endospore formation [17], and acid and gas production from glucose [18]. Physiological tests measured the growth of the isolates under different conditions of salt concentration [19], temperature [20], and pH [19]. Based on the results of these tests, the isolates were classified into different genera [17].

### 2.8. Physicochemical Analysis

Various physicochemical analyses in the study used different reference procedures found in various literature sources, for example, pH and temperature [21, 22], titratable acidity (TA) [22, 23], moisture and solid contents [22, 24], alcohol content [22, 25], nutritional value [22, 26], total protein content [22, 24, 27], total fat content [22, 24, 27], ash content [22, 24, 27], and total carbohydrate [22, 24].

### 2.9. Data Analysis

The data obtained from the respondents were analyzed using SPSS software version 20.0. The mean values and separation of microbial isolates of the *Jikita* samples from different sites were compared using a one-way ANOVA and ANOVA post hoc analysis. Differences were considered significant at a 95% confidence interval (*p* < 0.05).

## 3. Results

### 3.1. Sociodemographic Characteristics of *Jikita* Vendors

The data on the sociodemographic characteristics of *Jikita* vendors were collected after obtaining informed consent from the respondents. Jikita is a traditional fermented beverage made from a combination of cereal crops and sold by women. All the vendors surveyed were female, with the most common age group being 31–40 years (41.1%). In terms of education level, 38.2% had attended primary school, while 32.3% were illiterate. The majority of the vendors were married (32.3%), and 38.2% had been selling *Jikita* for 11–15 years.

### 3.2. Knowledge of *Jikita* Production

All 100% of the 34 people who participated in this study were well experienced with *Jikita* production and vending. The source of knowledge for *Jikita* productions was 61.8% obtained from family while 38.2% was obtained from relatives. All those who produce rely on the same type of raw materials for *Jikita* production: maize, barley, wheat, and malt flour. Accordingly, 70.7% of *Jikita* producers and vendors bought raw material from the market only, while 23.5% brought from both shop and market. Among the producers, 44.1% produced *Jikita* once a week and 32.3% produced twice.

### 3.3. Description of the Production Processes of *Jikita*

The basic processing steps of *Jikita* fermentations are similar, although every *Jikita* producer seems to have their own recipe based on tradition and the economic situation. The vat used for *Jikita* preparation was washed and cleaned using *Ebicha* leaves (*Vernonia amygdalina*). Then, the vat was smoked using *Ejersa* wood (*Olea africana*) and was ready for *Tinsis* preparation. One kilogram each of maize (*Zea mays*), malted barley (*Hordeum vulgare*), and wheat (*Triticum* sp.) was ground and mixed with 5 L of water in a vat called *Tinsis*. Seal the fermentation vat tightly and allow the mixture to ferment for 3–4 days.

**Figure 1:**
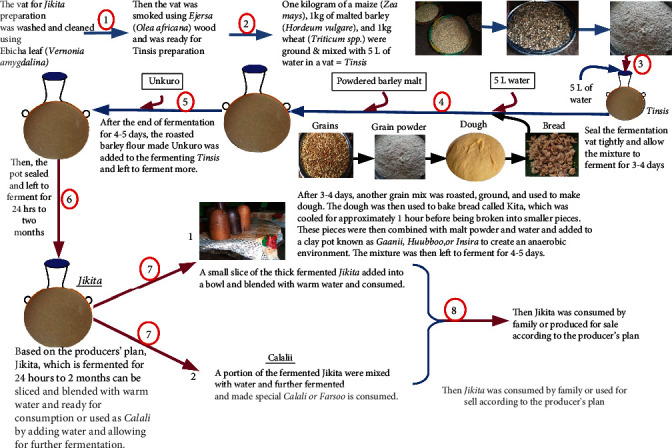
Traditional Jikita preparation procedure.

After 3–4 days, another grain mix was roasted, ground, and used to make dough. The dough was then used to bake bread called *Kita*, which was cooled for approximately 1 h before being broken into smaller pieces. These pieces were then combined with 1 kg of malt powder and 5 L of water and added to a clay pot known as *Gaanii*, *Huubboo*, or *Insira* to create an anaerobic environment. The mixture was then left to ferment for 4–5 days. After the fermentation period of 4–5 days, roasted barley flour made *Unkuro* (which is a roasted and ground grain powder that is moistened with a small amount of water and heated on a metal pan for 20–30 min) was added to the fermenting *Tinsis* and left to ferment further. Then, the pot was sealed and left to ferment for 24 h to 2 months. According to the producers' plan, *Jikita*, which is fermented for 24 h to 2 months (Figure 1), can be sliced and blended with warm water for consumption or used as *Calali* by adding water and allowing for further fermentation.

A small slice of the thick fermented *Jikita* is added to a bowl, blended with warm water, and consumed. A portion of the fermented *Jikita* is mixed with water, further fermented, and made into special *Calali/Farsoo* (a portion of fermented *Jikita* mixed with water in a separate pot called *Huubboo*), which is consumed. Finally, *Jikita* is either consumed by the family or sold, depending on the producer's plan.

### 3.4. Physicochemical Characteristics of *Jikita* Samples

The physical and chemical properties of the *Jikita* samples were tested and analyzed (Table 1).

Fermentation exerts an influence on various physicochemical parameters, encompassing acidity, moisture, and total solid content, characteristics commonly altered during microbial fermentation.  Table 2 presents comprehensive test outcomes, inclusive of mean values, standard deviations, and ranges of variation. Notably, the coefficient of variation (CV) for pH and moisture content registered less than 10%, whereas other tests exhibited a CV exceeding 10%.

### 3.5. Physicochemical Parameters and Proximate Composition of *Jikita* During Laboratory-Based Fermentation

This study analyzed the physical and chemical properties, as well as the proximate composition, of fermented *Jikita*. The study determined the pH, TA, temperature, moisture content, total solid content, total protein, total fat, total ash, and total carbohydrate. During the fermentation process, the pH of *Jikita* decreased from 4.15 ± 0.08 to 3.77 ± 0.02, while the TA and moisture content increased from 0.25 ± 0.07 to 0.57 ± 0.01 and 74.46 ± 0.37 to 81.29 ± 0.19, respectively. Additionally, the total solid content decreased from 25.5 ± 0.37 to 18.70 ± 0.19, as shown in Table 3.

### 3.6. Microbial Analysis

#### 3.6.1. Mean Microbial Count

In a study of 30 *Jikita* samples, microbial analysis involved homogenizing 10 g of sample with peptone water, followed by serial dilutions. Results indicated persistent molds and a dominance of aerobic mesophilic bacteria and spore formers (Table 3).

#### 3.6.2. Prevalence of *Staphylococcus aureus* and *Salmonella* sp. in *Jikita* Samples

In this study, 13 out of 30 samples (43.33%) were found to contain *Staphylococcus aureus*. Samples collected from both study site (Gedo and Babicha) districts showed the presence of this bacterium; however, *Salmonella* sp. was not detected in any of the *Jikita* samples.

#### 3.6.3. Microbial Dynamics of *Jikita* Fermentation

**Figure 2:**
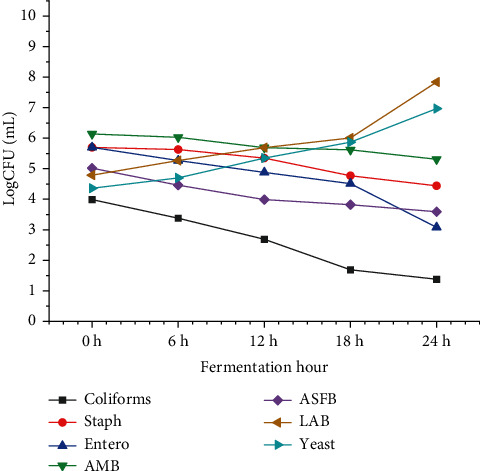
Microbial dynamics (mean ± SD) during Jikita fermentation.

In the laboratory, the microbial growth dynamics of the *Jikita* fermentation process were studied at 6-h intervals over a period of 24 h. The tests were performed at different times during the fermentation, such as 0, 6, 12, 18, and 24 h. The results showed that as the fermentation time increased, the number of LAB and yeast increased while the number of Enterobacteriaceae and coliforms decreased (Figure 2).

Therefore, fermentation has a positive impact on the growth of beneficial microorganisms while simultaneously decreasing the number of pathogenic microorganisms. These findings demonstrate the significant role that the duration of fermentation plays in microbial growth and product formation.

#### 3.6.4. Identification and Characterization of LAB

A total of 196 LAB were isolated and analyzed from fermented *Jikita* samples. The results of the biochemical characterization showed that all the isolates were Gram-positive, lacked catalase activity, were non–spore-forming, and were nonmotile. They were either rods, cocci, or cocci in tetrads. The classification of the isolates was based on morphological, biochemical, and physiological characteristics [17]. The results indicated that 45% of the isolates belonged to the *Lactobacillus* genus, 23.47% for *Lactococcus* sp., 18.37% for *Leuconostoc* sp., and 13.26% for *Enterococcus* sp. (Table 4).

#### 3.6.5. Macroscopic and Microscopic Colony Morphology of Yeast From *Jikita* Samples

##### 3.6.5.1. Macroscopic Morphology of Yeasts

A total of 255 yeast isolates were isolated from 30 *Jikita* samples, which were then analyzed for their macroscopic morphological features, including shape, color, size, elevation, texture, and margin. The majority of the yeast isolates were circular (94.5%) with some irregular shapes (5.5%).

In terms of color, 32.15% were white and 67.85% were creamy white. The size of the yeast isolates varied with 11.76% being small, 54.5% medium, and 33.7% large. The elevation of the yeast isolates was either raised (5.5%), pulvinate (11%), convex (16.8%), umbonate (63.9%), or flat (2.7%). All yeast isolates had a smooth texture, and the margin was either entire (98.8%) or undulate (1.17%).

##### 3.6.5.2. Microscopic Morphological Observation of Yeasts

The microscopic observation of yeast cells was focused on cell shape, size, budding, and the presence/absence of pseudohyphae. Results showed various shapes, including oval (36%), ovoid (28%), lemon-shaped (18.4%), and circular (17.6%). Sizes were classified as small (24.3%), medium (40%), or large (35.7%). With regard to budding, monopolar (49.8%), bipolar (15.3%), both mono- and bipolar (18%), or multilateral (16.9%) was observed. Additionally, the presence of pseudohyphae was seen in 26% of the cells, while 74% lacked pseudohyphae.

## 4. Discussion

The demographic data reveals that a significant proportion of *Jikita* vendors are women aged 31–40, which aligns with the cultural perception of *Jikita* preparation as a female-oriented task. This is comparable to the gender roles observed in *Bukuri* preparation in East Wollega, as noted by [28]. The educational background of the majority of vendors, who have only completed primary education, suggests that *Jikita* vending may be an accessible livelihood for those with limited formal education. Furthermore, the marital status of the vendors, with 32.3% being married, indicates that Jikita vending serves as a viable economic activity for individuals in various life stages. The low capital requirement for starting a *Jikita* business makes it an attractive option for those looking to enter the market with minimal investment.

During the study, 34 women who were experienced in preparing *Jikita* were interviewed. They emphasized the importance of family and relatives in the transfer of knowledge, highlighting the crucial role of social networks in preserving traditional food practices. The use of ingredients such as maize, barley, wheat, *Tef*, and malt barley flour, predominantly sourced from the open market, reflects the local agricultural produce and the vendors' reliance on market availability.

Variations in *Jikita* preparation frequency, ranging from once to thrice weekly, may reflect the demand dynamics and the flexibility of the vendors to adapt to market needs. The unanimous lack of special clothing usage among vendors, a stark contrast to the 42.86% reported by Chukuezi [29] in Owerri, Nigeria, raises questions about hygiene standards and customer perceptions in the food vending industry. Overall, these insights provide a deeper understanding of the socioeconomic factors influencing *Jikita* vending and offer a basis for further research into the implications of these practices on public health and local economies.

The average total count of AMB (6.13 log CFU/mL) in this study is consistent with the findings of Kitessa et al. [30], who reported > 3 log CFU/mL of AMB in Shameta collected from the homes of lactating mothers. The *Jikita* sample had the same AMB load as the standard count [31]. The presence may be due to inadequate education, improper cleaning of vending materials, the use of unsafe water to dilute the ready-to-use *Jikita*, unsanitary utensils, and dust and litter in the vending area. Shamebo, Bacha, and Ketema [32] identified low education levels, hygiene issues, and a lack of awareness of contamination as key factors contributing to high counts of AMB.

Besides that, the average count of Enterobacteriaceae was 4.9 log CFU/mL, while the average count of coliforms was 2.79. These results are consistent with the microbial dynamics of *Borde* fermentation as reported by Nemo and Bacha [22]. According to Nemo and Bacha, at the start of the fermentation process, aerobic mesophilic bacteria, staphylococci, and Enterobacteriaceae initiate the process with a count of over 5 logs CFU/mL. The presence of these microorganisms may be attributed to inadequate hand hygiene practices after using the toilet. Ahimed and Sirag [33] discovered that enteric microorganisms were present due to fecal contamination caused by improper hand washing after using the toilet. Nworie et al. [34] also noted that the presence of Gram-negative bacteria, such as coliforms, indicates a likelihood of fecal contamination in public restrooms.

The average staphylococcal count in the present study was 3.29 log CFU/mL, which is consistent with the findings of Kitessa et al. [30] regarding the *Shameta* sample collected from households of lactating mothers, which is greater count than FAO standard for ready-to-eat food [35]. In their study, the staphylococcal count ranged between 2 and 3.28 log CFU/mL. The higher staphylococcal count observed in our study may be attributed to the unhygienic handling of *Jikita* by producers and vendors, as well as inadequate personal hygiene. Interviews conducted with *Jikita* producers and vendors revealed that 100% of them did not use special garments during *Jikita* production. It is worth noting that staphylococci can be present in the air, dust, skin, sewage, water, and food, as well as on equipment and environmental surfaces.

Contrary to the report by Nemo and Bacha [22] on aerobic spore-forming bacteria (ASFB) (*Bacillus* sp.) in border microbial dynamics, which stated a mean count of > 3 log CFU/mL, this current study reports a higher mean count of ABSF bacteria (5.4 log CFU/mL). The presence of ASFB in Jikita beverages could be attributed to the ingredients and processing methods used. These aerobic spore farmers secrete a variety of degradative enzymes, such as amylases and proteases, which play a crucial role in maintaining a supply of simple sugars for fermentation processes to continue [36]. While aerobic spore formers can contribute to fermentation, they are unable to do so in the presence of sufficient numbers of LAB, as aerobic mesophilic bacteria cannot multiply at low pH [1].

The average count of LAB in this study was 5.83 log CFU/mL, which is lower than the count reported by Chali, Anbessa, and Ketema [28] in *Bukuri*, where counts ranged from 5.05 to 7.74 log CFU/mL. Having a high concentration of LAB in beverages is significant because it can help reduce levels of pathogens. These bacteria have the ability to produce antimicrobial compounds (*lactic acid*, *bacteriocins*, *hydrogen peroxide*, and *antifungal peptides*) that can fight against competing flora, including food-borne spoilage and pathogenic bacteria [37]. A high count of LAB could be responsible for acidifying the product as fermentation periods are expanded. LAB has been found to play a role in the natural fermentation of many traditional Ethiopian fermented foods and beverages [38]. The low count of LAB in this study could be attributed to the ingredients used for fermentation and environmental factors, such as temperature and moisture content [36, 39].

*Staphylococcus* sp. accounted for 39.05% of the bacterial isolates in this study, making it the most common contaminant. It was followed by *Micrococcus* sp. (17.15%), *Bacillus* sp. (15.33%), and Enterobacteriaceae (14.96%), *Pseudomonas* sp. (5.47%), *Alcaligenes* sp. (4.74%), *Acinetobacter* sp. (2.2%), and *Providencia* sp. (1.1%). The high prevalence of *Staphylococcus* sp. can be attributed to its presence as a major component of the skin and nasal flora, as well as its ability to easily spread through various human activities.

The mean pH of Jikita samples from the Gedo and Babicha districts in the current study was 3.67 ± 0.09, indicating that it was generally consistent with Kitessa et al.'s report from 2022. Kitessa et al. stated that the pH values in maize-based *Shameta* samples from Guto Gida, East Wollega Zone, were 3.6 ± 0.02. The low pH values of *Jikita* and other fermented beverages may be due to the metabolites produced by LABs during the fermentation process. According to [38], *Jikita* may contain specific metabolites that could cause a decrease in pH and lead to the formation of organic molecules such as acetic acid and propanoic acid, which are produced by LAB strains. These factors may also contribute to the acidic character of the samples.

In the study of microbial growth dynamics of laboratory-prepared *Jikita* fermented beverage, the early-stage fermentation was dominated by microbial counts (> 5 log CFU/mL) of AMB, Enterobacteriaceae, ASFB, and *Staphylococcus*. However, yeast and LAB were dominant at the end of fermentation. Yeast and LAB grow faster because they possess the necessary mechanisms to survive in an acidic environment. However, *Salmonella* spp. were not detected in this work, which is similar to WHO/FAO [35]. The microbial dynamics observed were consistent with those of Nemo and Bacha [22] for *Borde* microbial dynamics. At the start of AMB fermentation, *Staphylococcus* and Enterobacteriaceae initiated the fermentation process (> 5 log CFU/mL), but yeasts and LAB took over and dominated the fermentation process until the end.

The mean pH value of *Jikita* prepared under laboratory fermentation decreased from 4.15 ± 0.08 to 3.77 ± 0.02 from the beginning to the end of fermentation. In contrast, the TA and moisture content of *Jikita* fermentation increased as fermentation time increased, from 0.25 ± 0.07 to 0.57 ± 0.01 and from 74.46 ± 0.37 to 81.29 ± 0.19, respectively. The total solid content decreased from 25.5 ± 0.37 to 18.70 ± 0.19. The increase in moisture content can be attributed to the addition of water to the substrate before fermentation [40]. The increment of moisture content with fermentation time is the result of the consumption of dry matter and water production during aerobic and anaerobic catabolism by yeasts and LAB [41].

## 5. Conclusion

This study was the first to analyze the microbial, physicochemical, and nutritional properties of *Jikita*, a traditional Ethiopian fermented beverage. The results showed that *Jikita* is mainly produced and sold by women and serves various social and economic functions. The fermentation process involves a complex microbial community, dominated by *Staphylococcus* sp., *Micrococcus* sp., *Bacillus* sp., and Enterobacteriaceae. These microorganisms may pose a health risk due to their potential pathogenicity. However, fermentation also enhances the nutritional value of *Jikita* by increasing the levels of LAB and yeast, which have beneficial effects on human health and inhibit the growth of undesirable microbes. Furthermore, investigation is needed to determine the optimal fermentation time and conditions for *Jikita* production, as overfermentation can result in excessive sourness and spoilage. The study also revealed that molds are rarely present in *Jikita*, except in some samples that were exposed to air or contaminated by other sources. These findings provide valuable insights into the microbiology, chemistry, and nutrition of *Jikita*, as well as its cultural and economic significance. They also suggest directions for future research on improving the quality and safety of this traditional fermented beverage.

## Figures and Tables

**Table 1:** Physicochemical characteristics of *Jikita* samples collected from *Jikita* sellers in Gedo and Babicha districts, West Shewa Zone.

**Parameter**	***N***	**M** **e** **a** **n** ± **SD**	**Maximum**	**Minimum**	**%CV**
pH	30	3.67 ± 0.09	3.90	3.5	2.45
Titratable acidity		0.36 ± 0.10	0.49	0.13	27.77
Moisture content		76.42 ± 3.02	81.20	70.3	3.95
Total solid content		23.23 ± 2.85	28.77	18.8	12.26
Alcohol content		5.22 ± 0.74	6.73	4.20	14.1

Abbreviations: CV = coefficient of variation, *N* = number of samples analyzed, SD = standard deviation.

**Table 2:** Physicochemical parameters and proximate composition of Jikita during laboratory-based fermentation (mean ± SD).

**DF (h)**	**pH**	**T°C**	**TA**	**MC**	**TSC**	**TP**	**TF**	**T ash**	**TCHO**
0	4.15 ± 0.08	22.67 ± 0.41	0.25 ± 0.07	74.46 ± 0.37	25.5 ± 0.37	18.48 ± 0.01	0.97 ± 0.01	1.75 ± 0.00	4.34 ± 0.01
6	4.01 ± 0.02	24.23 ± 0.25	0.36 ± 0.01	75.38 ± 0.22	24.61 ± 0.22	16.68 ± 0.00	1.5 ± 0.00	1.26 ± 0.01	5.18 ± 0.01
12	3.93 ± 0.00	21.13 ± 0.20	0.42 ± 0.03	77.35 ± 0.23	22.64 ± 0.23	17.58 ± 0.00	1.56 ± 0.01	1.30 ± 0.01	2.21 ± 0.01
18	3.78 ± 0.01	22.06 ± 0.25	0.54 ± 0.00	78.99 ± 0.47	20.94 ± 0.49	19.70 ± 0.00	1.65 ± 0.01	1.73 ± 0.01	2.07 ± 0.01
24	3.77 ± 0.02	21.96 ± 0.23	0.57 ± 0.01	81.29 ± 0.19	18.70 ± 0.19	12.01 ± 0.00	1.68 ± 0.01	2.11 ± 0.01	2.91 ± 0.01

Abbreviations: DF = duration of fermentation, MC = moisture content, T°C = temperature, TA = titratable acidity, T ash = total ash, TCHO = total carbohydrate, TF = total fat, TP = total protein, TSC = total solid content.

**Table 3:** Mean microbial count (log CFU/milliliter) of *Jikita* samples collected from Gedo and Babicha districts, West Shewa Zone.

**Microbial groups**	**Sample size**	**M** **e** **a** **n** ± **Std**.**deviation**	**Minimum**	**Maximum**	**CV (%)**
AMB	30	6.13 ± 0.77	4.9	7.6	12.56
Enterobacteriaceae		4.99 ± 0.74	3.4	6.54	14.82
Coliforms		2.79 ± 0.37	2.28	3.48	8.78
ASFB		5.40 ± 0.70	4.25	6.74	12.96
*Staphylococcus* sp.		3.29 ± 0.49	2.2	5.32	14.89
LAB		5.83 ± 0.62	4.7	7.17	10.63
Yeast		5.98 ± 0.51	5.13	7.75	8.52
Mold		2.67 ± 1.18	0	4.13	44.2

Abbreviations: AMB, aerobic mesophilic bacteria; ASFB, aerobic spore-forming bacteria; LAB, lactic acid bacteria.

**Table 4:** Morphological, biochemical, and physiological characteristics of LAB isolated from *Jikita* samples.

**Group of LAB**				
	**I (** **n** = 36**)**	**II (** **n** = 88**)**	**III (** **n** = 26**)**	**IV (** **n** = 46**)**
Characteristics				
Cell morphology	Spherical or ovoid	Rod	Spherical or ovoid	Spherical or ovoid
Gram staining	+	+	+	+
Shape	Cocci	Rod	Cocci	Cocci
Arrangement	Singly or in pair	Pairs, short chains	Pairs, short chains	Singly or in chains
Catalase	−	−	−	−
KOH	−	−	−	−
Endospore	−	−	−	−
Motility	−	−	−	−
O/F	F	F	F	F
Sugar fermentation				
Lactose	+	+	+	+
Sucrose	+	+	+	+
Fructose	+	+	+	+
Maltose	+	+	+	+
Mannitol	+	+	+	+
NaCl tolerance				
2%	+	+	+	+
4%	+	+	+	+
6.50%	+/−	+/−	+	+/−
8%	−	−	−	−
10%	−	−	−	−
pH tolerance				
4.5	+	+	+	+
9.6	+	+/−	+	+/−
T°C tolerance				
15	+	−/+	+	+/−
45	+/−	+/−	+	−
Gas production from glucose	++	+/−	−	−
Likely genera	*Leuconostoc* sp.	*Lactobacillus* spp.	*Enterococcus* sp.	*Lactococcus* sp.

Abbreviations: −/+ = more than 80% did not grow, +/− = more than 80% grew, + = growth/or positive test result, − = no growth/or negative test result.

## Data Availability

The data used to support the findings of this study are included within the article.
